# A dataset of three vine water status indicators, weather records and soil available water capacity components collected from a rain-fed Mediterranean vineyard

**DOI:** 10.1016/j.dib.2025.111295

**Published:** 2025-01-23

**Authors:** Yulin Zhang, Léo Pichon, Bruno Tisseyre

**Affiliations:** ITAP, Univ. Montpellier, INRAE, Institut Agro, 2 Place Pierre Viala, 34060 Montpellier, France

**Keywords:** Vitis vinifera, Time series, Vine shoot growth index (iG-Apex), Predawn leaf water potential (Ψpd), Carbon isotope discrimination (δ13C), cv. Syrah, cv. Grenache noir

## Abstract

This dataset contains three key indicators of vine water status: vine shoot growth index (iG-Apex), predawn leaf water potential (Ψpd), and carbon isotope ratio (δ13C). Additionally, it includes weather data and soil measurements. Spatial data files of studied fields, plots, and some vines’ locations are also provided.

The data were collected from a rain-fed vineyard in Southern France, 4 km north of the Mediterranean Sea. Measurements were made at the plot level, with each plot consisting of 10 adjacent grapevines (Vitis Vinifera). The iG-Apex was recorded weekly from June 10 to August 22, 2022, across 70 vine plots. Ψpd was measured weekly between 3 a.m. and 5 a.m. in 12 of these plots using a pressure chamber. On August 23, 2022, 100 berries were sampled from each plot, and 1.5 mL of grape juice was extracted for δ13C analysis using a carbon analyzer and mass spectrometer. The dataset contains 761 iG-Apex measurements (70 time series), 720 Ψpd measurements (60 time series), and 70 single-date δ13C measurements. Weather data were recorded daily in 2022 from a weather station located at the vineyard's center, providing five parameters: cumulative rainfall, relative humidity, and three air temperatures (mean, minimum, and maximum). Soil available water capacity components (horizon thickness, field capacity, permanent wilting point, bulk density, and rock fragment content) were measured in 5 of the 70 plots after prior soil profile wall analyses.

Monitoring vine water status is essential for optimizing grape yield and wine quality. While Ψpd and δ13C are considered reference methods, they are expensive and prone to logistical constrains. In contrast, iG-Apex can be collected with minimal time and financial investment. This dataset enables not only the exploration of statistical relationships between the plant-based and soil-based indicators, but also the modeling of Ψpd and/or δ13C using iG-Apex, while accounting for weather and soil influences. All data were georeferenced, allowing future integration of ancillary spatial data sources, like multi-spectral remote sensing images or yield data.

Specifications Table

**Table:** 

Subject	Agronomy and Crop Science
Specific subject area	The relationship between vine water status indicators and their different sensitivities towards weather and soil conditions
Type of data	Table files (.csv) Spatial data files (.gpkg)
Data collection	The dataset was collected from a rain-fed vineyard in Southern France in 2022. The vine shoot growth index (iG-Apex) was measured weekly from June to August by one operator using the smartphone application ApeX-Vigne, across 70 vine plots. Predawn leaf water potential (Ψpd) was measured in 12 of these plots between 3 a.m. and 5 a.m., on the same dates as iG-Apex measurements. This task was performed by two operators using a pressure chamber. Additionally, before harvest, grape juice samples (1,5 mL) were collected from all 70 plots, and the carbon isotope ratio (δ13C) was analysed in the laboratory. Daily weather data were provided by a weather station that was located in the center of the vineyard. In 5 studied plots, soil profile analyses were conducted to identify soil horizons. Samples from each horizon were collected to evaluate soil properties which allows the calculation of soil available water capacity in a given plot. The measurement locations were georeferenced using a high precision GNSS RTK (Global Navigation Satellite System Real-Time Kinematic) system.
Data source location	Institution: Institut Agro Montpellier City: Villeneuve-lès-Maguelone Country: France Latitude and longitude: 43.547417; 3.8414769
Data accessibility	Repository name: Zenodo Data identification number: 10.5281/zenodo.13992359 Direct URL to data: 10.5281/zenodo.13992359
Related research article	Zhang, Y., Pichon, L., Pellegrino, A., Roux, S., Péruzzaro, C., Tisseyre, B., 2024. Predicting predawn leaf water potential while accounting for uncertainty using vine shoot growth and weather data in Mediterranean rainfed vineyards. Agricultural Water Management, Volume 302, 108998. 10.1016/j.agwat.2024.108998

## 1 Value of the Data

• *Potential for modeling*: This dataset enables the exploration of statistical relationships between three key vine water status indicators. It supports the development of predictive models that use easily collected data, such as the iG-Apex or weather information, to estimate more resource-demanding measures like Ψpd or δ13C. By modeling these relationships, the dataset can help overcome logistical constraints in monitoring vine water status in commercial vineyards. In parallel, soil available water capacity data could be used as input parameters in crop mechanistic models, which will allow accurate model parameterization, thereby assisting the validation of mechanistic models at a local scale. Soil data can equally be used to explain variabilities observed in plant-based water status indicators.
• *Potential for sensitivity analysis*: The dataset offers an opportunity to assess how weather and soil factors influence the temporal and spatial variability of vine water status. It allows researchers to quantify the impact of climate on the indicators over time and examine how soil conditions contribute to their spatial variability. Data users can equally explore how these environmental factors affect differently each of the three water status indicators.
• *Potential for integrating ancillary data sources*: All measurement plots, including vines used for Ψpd measurements, are georeferenced with centimetric precision. This spatial accuracy allows future users to link additional agricultural data sources—such as satellite images, yield [1] or vine density data [2] —to the locations provided in this dataset. This integration of various data types can enhance potentials in modeling and improve sensitivity analyses.

## 2 Background

Monitoring vine water status is time-consuming and expensive. The primary objective of this dataset was to explore the potential of iG-Apex, an easily collectable data source, for developing a predictive model of Ψpd, aimed at simplifying the monitoring process for viticultural professionals [3]. In the associated research paper, iG-Apex, Ψpd, and weather data were integrated into a predictive model. However, soil information, such as the soil parameters included in this dataset, which are used to calculate soil available water capacity (SAWC), was not part of the original model. SAWC is known to significantly influence vine water status [4]. Additionally, δ13C has shown interesting correlations with SAWC [5], offering a potential less restrictive alternative for incorporating soil-related information into iG-Apex-based water status models. Additionally, it is equally possible to conceive methods for estimating the value of SAWC by using plant-based water status indicators, like iG-Apex, because the latters are generally easier to collect in field. Lastly, the spatial coordinates of measurement plots provided in this dataset facilitate the integration of other data sources, such as multi-spectral satellite images, for future model improvements.

## 3 Data Description

**Fig. 1:**
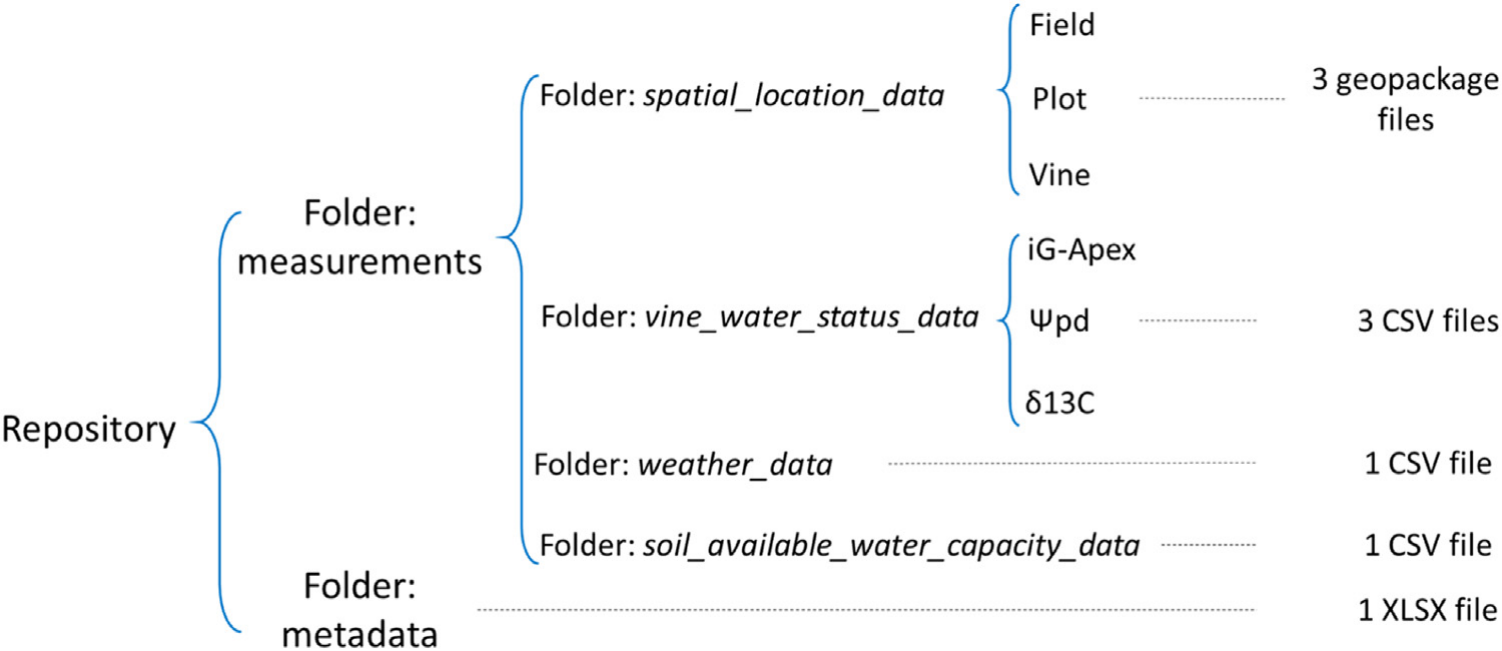
Structure of the data repository.

The dataset is stored in a data repository (Fig. 1), supplemented by a metadata file: “metadata.xlsx” which contains additional information for easing the use of the dataset.

The dataset included 4 types of data:

• **Spatial location data**: Data were collected from five fields, numbered 1 through 5. The geopackage file “field_contour.gpkg” contains 5 spatial polygons representing the contours of these fields. Field information was summarized in Table 1.

**Table 1:** Description of variables in the geopackage data file “field_contour.gpkg”.

Variable	Description	Unit
Fid	Identifier of a spatial object which was generated automatically during creation	unitless
FIELD	Field number	unitless
NAME	Name of a field used in the vineyard	unitless
CULTIVAR	Grapevine's cultivar name in a given field	unitless
AREA	Area of a field	ha
DIST_BETWEEN_ROWS	Distance between two rows of vines in a given field	m
DIST_BETWEEN_VINES	Distance between two vines of vines in a given field	m
  Within each field, measurement plots were numbered from 1 to N, with the total number of plots summing to 70 across all fields. Each plot consisted of 10 vines. The spatial boundaries of a plot were defined by the first and last vines. Depending on its location, a plot spanned either one or two rows, requiring either two or four vines to define the spatial boundaries. Therefore, the geopackage file “plot_location_polygons.gpkg” contains 70 spatial polygons corresponding to 70 plots, with 18 of them located in one row and 52 located in two rows. In 12 of these plots, Ψpd measurements were conducted on five specific vines, numbered from 1 to 5. The geopackage file “plwp_measurement_plots.gpkg” contains 60 spatial points representing these individual vines (12 plots × 5 vines) used for Ψpd measurements. All spatial objects were associated with the number of a field, and when applicable, those of plot and vine.
• **Vine water status data**: The vine shoot growth index (iG-Apex), predawn leaf water potential (Ψpd), and carbon isotope ratio (δ13C) are contained in three separate table files: “vine_shoot_growth_index.csv,” “predawn_leaf_water_potential.csv,” and “carbon_isotope_ratio.csv,” respectively. Each measured value was identified by a number of field and plot. iG-Apex was measured for each of the 70 plots on 8 to 12 dates, resulting in 761 rows of data. In each plot and for each date, the counts of full growth (FG), moderate growth (MG), and stopped growth (SG) vine shoots were provided alongside the iG-Apex readings. In 12 of the 70 plots, Ψpd measurements were taken on the same dates as iG-Apex observations, with 5 Ψpd readings per plot per date, as Ψpd was measured at the individual vine level (and identified by a vine number). This consists of 720 rows of Ψpd data. For δ13C, a single value was obtained for each of the 70 plots on August 23, 2022, giving 70 rows of data. All variables and their units in these three table files are summarized in Table 2.

**Table 2:** Description of variables in files containing vine water status data.

Variable	Description	Unit
FIELD	Field number (present in each table)	unitless
PLOT	Number of plot in a given field (present in each table)	unitless
VINE	Number of a vine in a given plot (present only in table “predawn_leaf_water_status.csv”)	unitless
DATE	Date of a measurement (present in each table)	DD/MM/YYYY
NB_FG	Count of vine shoots at the Full Growth (FG) stage (When the last two unfolded leaves are folded along the axis of the shoot, they do not cover it; present only in table “vine_shoot_growth_index.csv”)	unitless
NB_MG	Count of vine shoots at the Moderate Growth (MG) stage (When the last two unfolded leaves are folded along the axis of the shoot, they cover it; present only in table “vine_shoot_growth_index.csv”)	unitless
NB_SG	Count of vine shoots at the Stopped Growth (SG) stage (When the vine shoot is dry or cracked; present only in table “vine_shoot_growth_index.csv”)	unitless
iG-Apex	The index of vine shoot growth. It is calculated as an average of weights attributed to the 50 observed vine shoots. The weights depend on whether each observed vine shoot is classified as category FG, MG, or SG. (present only in table “vine_shoot_growth_index.csv”)	unitless
PLWP	Predawn Leaf Water Potential (PLWP/Ψpd) is measured using a pressure chamber [6]. The potential value is noted when moisture just begin to appear on the section of the woody bundle of the observed petiole. Its sign is systematically negative, with lower values indicating more severe water deficit levels. (present only in table “predawn_leaf_water_status.csv”)	MPa
DELTA_C13	Carbon isotope ratio (δ13C) is calculated from the carbon isotopes 13C and 12C content in berries. It is an overall indicator of the water stress suffered by the vine during the ripening period. Its sign is negative, with higher values indicating more severe water deficit levels. (present only in table “carbon_isotope_ratio.csv”)	‰
• **Weather data**: The data were recorded by a weather station located in the center of the vineyard (close to Field No. 4). The daily weather variables include rainfall, relative humidity, mean air temperature, maximum air temperature, and minimum air temperature. The data are provided in a table file: “weather_variables.csv”, containing 365 rows (from January 1 to December 31, 2022). Descriptions and units of these variables are provided in Table 3.

**Table 3:** Description of variables in “weather_variables.csv”.

Variable	Description	Unit
FIELD	Field number	unitless
DATE	Date of a measurement	DD/MM/YYYY
RAINFALL	Amount of precipitation that fell during 24 hours	mm
RH	Average relative humidity during 24 hours	%
AIR_TEMP_MEAN	Average air temperature during 24 hours	°C
AIR_TEMP_MAX	Maximum air temperature during 24 hours	°C
AIR_TEMP_MIN	Minimum air temperature during 24 hours	°C
• Soil available water capacity data: Soil profile wall analysis was conducted in 5 plots across 2 fields, with soil horizons identified prior to sampling and coded by letters. In each horizon, the following soil parameters were measured either in the field or through laboratory analysis: horizon thickness, field capacity, permanent wilting point, bulk density, and rock fragment content. Then, the soil available water capacity (SAWC) of each horizon was computed by using these parameters. The data are provided in a table file: “soil_available_water_capacity.csv”, containing 21 rows, with each row representing the measurements for a specific horizon. Explanations and units of these variables are detailed in Table 4.

**Table 4:** Description of variables in “soil_available_water_capacity.csv”.

Variable	Description	Unit
FIELD	Field number	unitless
PLOT	Number of a plot in a given field	unitless
HORIZON	Code of a horizon in a given plot. The order of letters indicates the increasing soil depth.	unitless
THICKNESS	Thickness of a horizon measured in field.	dm
FIELD_CAPACITY	Field capacity water content quantifies the gravimetric soil water content held after rapid drainage.	g.g^−1^
PERM_WILT_POINT	Permanent wilting point quantifies the gravimetric soil water content which causes an irreversible leaf wilt due to the lack of water.	g.g^−1^
BULK_DENSITY	Bulk density is the ratio between the dry mass of soil and its volume.	g.cm^−3^
ROCK_FRAGMENT	Rock fragment is the gravimetric ratio of the dry mass of coarse particles (with a diameter greater than 2 mm) to the total dry mass of the soil, including these coarse elements.	g.g^−1^
SAWC	Soil available water capacity is calculated for each identified horizon, which quantifies the maximum volume of water the horizon can hold and useable for the plant's rooting system.	mm

Equation 1:

$$\begin{array}{ccc} \text{SAWC} & = & \;\sum_{i=1}^{i=\text{maxNbHorizon}}\text{THICKNES}S_{i}\times \text{BULK}\_\text{DENSIT}Y_{i}\times \left(\text{FIELD}\_\text{CAPACIT}Y_{i}-\;\text{PERM}\_\text{WILT}\_\text{POIN}T_{i}\right)\\ & & \times \left(1-\text{ROCK}\_\text{FRAGMEN}T_{i}\right) \end{array}$$

## 4 Experimental Design, Materials and Methods

### 4.1 Experimental fields and plots

**Fig. 2:**
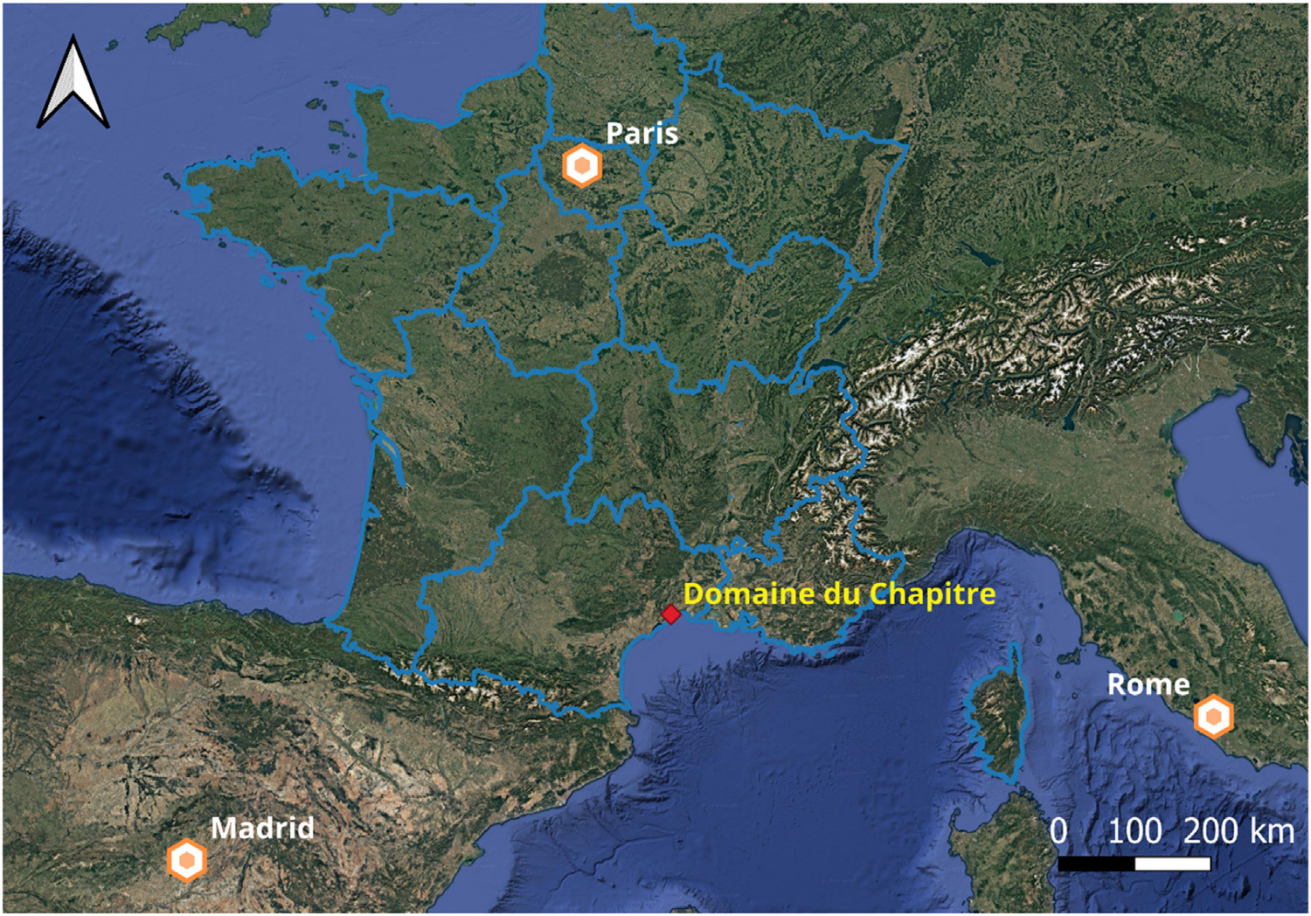
Location of the vineyard “Domaine du Chapitre” on the Mediterranean coast in Southern France.

The dataset was collected from a 30-hectare rain-fed vineyard located in Southern France (“Domaine du Chapitre,” Villeneuve-lès-Maguelone; 43.532300° N, 3.864230° E; Fig. 2). The region's climate is predominantly warm and temperate, with hot, dry summers [7]. The vineyard's soils are generally deep (> 1 m) and primarily calcisols and cambisols, characterized by a high silt content, as described in detail by [1]. Vines’ training method was characterized by vertical shoot positioning with 3 levels of trellising, with canopy regulation during the vegetative development stage. The plant density varies between 3300 and 4000 vines/ha.

**Fig. 3:**
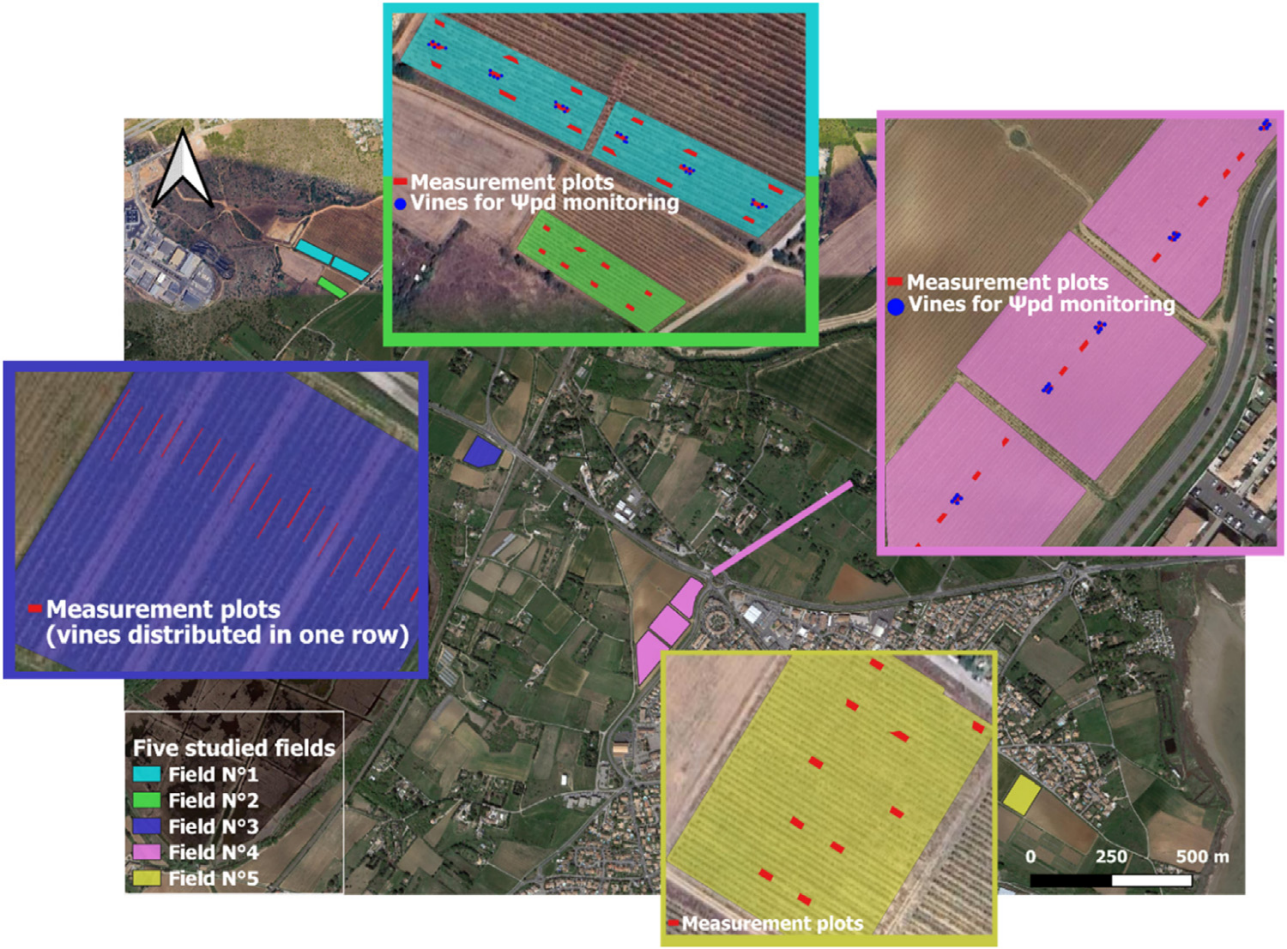
Position of studied fields and location of the 70 measurement plots (with some of them containing vines used for predawn leaf water potential measurements).

Four fields (N°1-4) contained Vitis vinifera L. cv. Syrah, while field N°5 contained cv. Grenache Noir. Plot locations were distributed evenly within each field to enhance the likelihood of capturing soil variability. Each plot included 10 neighboring, mature, non-senescing vines that were disease-free and unaffected by anomalies at the beginning of the growing season, and represented by a spatial polygon (Fig. 3). In field N°3, all 10 vines in a plot were in the same row, while in the remaining plots, they were distributed across two adjacent rows. The vines selected for Ψpd measurements were georeferenced using a real-time kinematic (RTK) GNSS receiver. Additionally, a static RTK base [8] was installed near the winery, at a maximum distance of 1.5 km from any of the studied field, allowing a centimetric precision. The WGS84 global coordinate reference system was used to represent latitude and longitude.

### 4.2 Vine shoot growth index (iG-Apex)

**Fig. 4:**
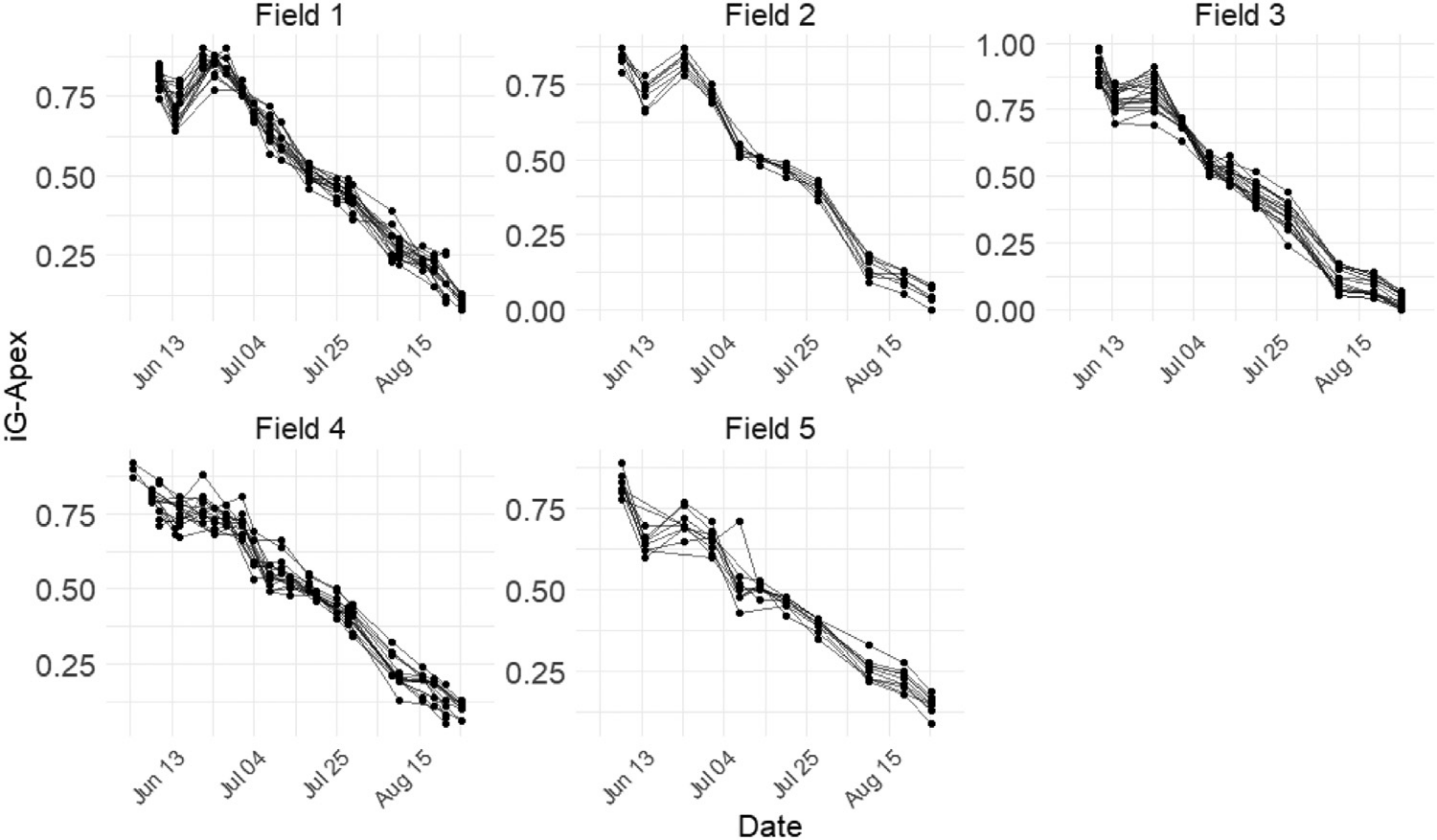
Temporal evolution of iG-Apex in 2022 in all 70 monitored plots for the 5 fields of the experiment.

The iG-Apex was collected using the smartphone application ApeX-Vigne [9]. In each measurement plot, an operator observed 50 vine shoots (5 per vine) and classified them into three categories: (a) Full Growth, (b) Moderate Growth, and (c) Stopped Growth. The iG-Apex was calculated as the average of weights assigned to these 50 shoots, with values of 1, 0.5, or 0, corresponding to classifications (a), (b), or (c), respectively. The smartphone application registered the numbers of shoots in each category, calculated iG-Apex, and stored the data. Measurements were taken weekly, whenever possible, between June 10 and August 22, 2022, across all 70 vine plots. In total, 70 time series of iG-Apex were obtained at the plot level (Fig. 4).

### 4.3 Predawn leaf water potential (Ψpd)

**Fig. 5:**
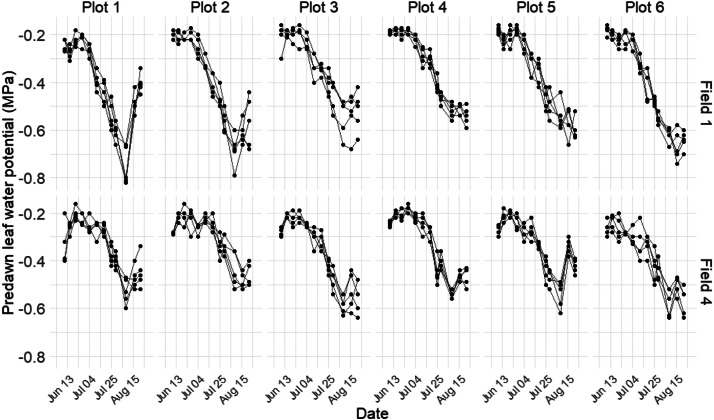
Temporal evolution of Ψpd in 2022 of the 60 monitored vines inside 12 plots.

Predawn leaf water potential (Ψpd) was measured between 3 a.m. and 5 a.m. using a pressure chamber [6]. In each measurement plot, 5 vines were selected, marked, and georeferenced using a RTK receiver. For each measurement, one fully developed, healthy leaf located in the middle of each vine's shoot was selected. The measurement was recorded when small drops of water just began to appear on the cut petiole as pressure was increased. This protocol was applied consistently across the 5 marked vines per plot, resulting in 5 Ψpd readings per plot for each measurement date. Two operators conducted all the measurements. Ψpd was monitored weekly in each plot, in alignment with the iG-Apex measurement schedule. A total of 60 (12 plots * 5 vines/plot) time series of Ψpd were obtained at the vine level and illustrated by Fig. 5.

### 4.4 Carbon isotope ratio (δ13C)

**Fig. 6:**
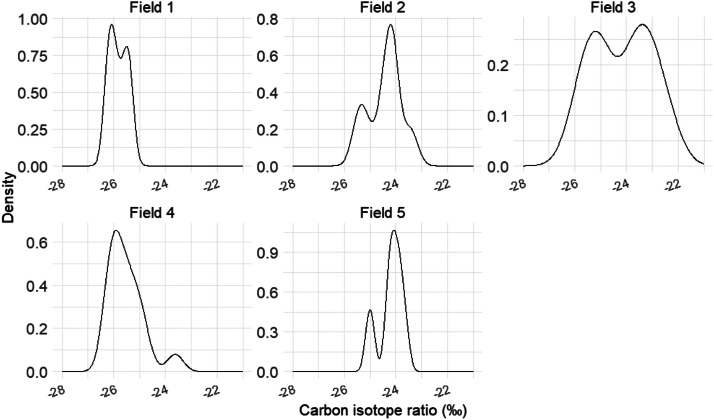
Distribution of δ13C observed values, from 70 studied plots over the 5 fields.

The δ13C measurement was performed on sugars from the ripening must (grape juice) sampled from each plot. On August 23, 2022, 100 berries (approximately 10 per vine) were collected from each of the 70 plots and placed into clean, rigid plastic bags. The berries were crushed in each bag, and 1.5 mL of grape juice was extracted and stored in microtubes at −18 °C until analysis. In the laboratory, the analysis was carried out using a carbon analyzer and mass spectrometer by Isotopes Quantifications platform (AQGI, Institut Agro Montpellier, France). δ13C values were expressed relative to the international Pee Dee Belemnite (PDB) standard [10]. A total of 70 single-date δ13C measurements were obtained across 5 fields, and the distribution of values in each field were presented in Fig. 6.

### 4.5 Weather data

The weather data were obtained from a commercial weather station, with detailed parameter settings, sensor types, and user interface described by [1]. Their dataset included weather variable readings recorded every 15 min. For this dataset, those variables were aggregated to a daily scale because this frequency is commonly used by professionals for vine water status monitoring. The aggregation operations used to derive the daily variables are detailed in Table 5. The data cover the period from January 1 to December 31, 2022, and are considered representative of all five studied fields due to their geographical proximity and the flat topography of the area.

**Table 5:** Aggregation operations to compute the daily weather variables.

Weather variable	Aggregation function
Rainfall	**Sum** of all 15-min readings over 24 h for a given date
Relative Humidity	**Average** of all 15-min readings over 24 h for a given date
Mean Air Temperature	
Maximum Air Temperature	**Highest** value recorded among all 15-min readings over 24 h for a given date
Minimum Air Temperature	**Lowest** value recorded among all 15-min readings over 24 h for a given date

### 4.6 Soil available water capacity (SAWC) components

**Fig. 7:**
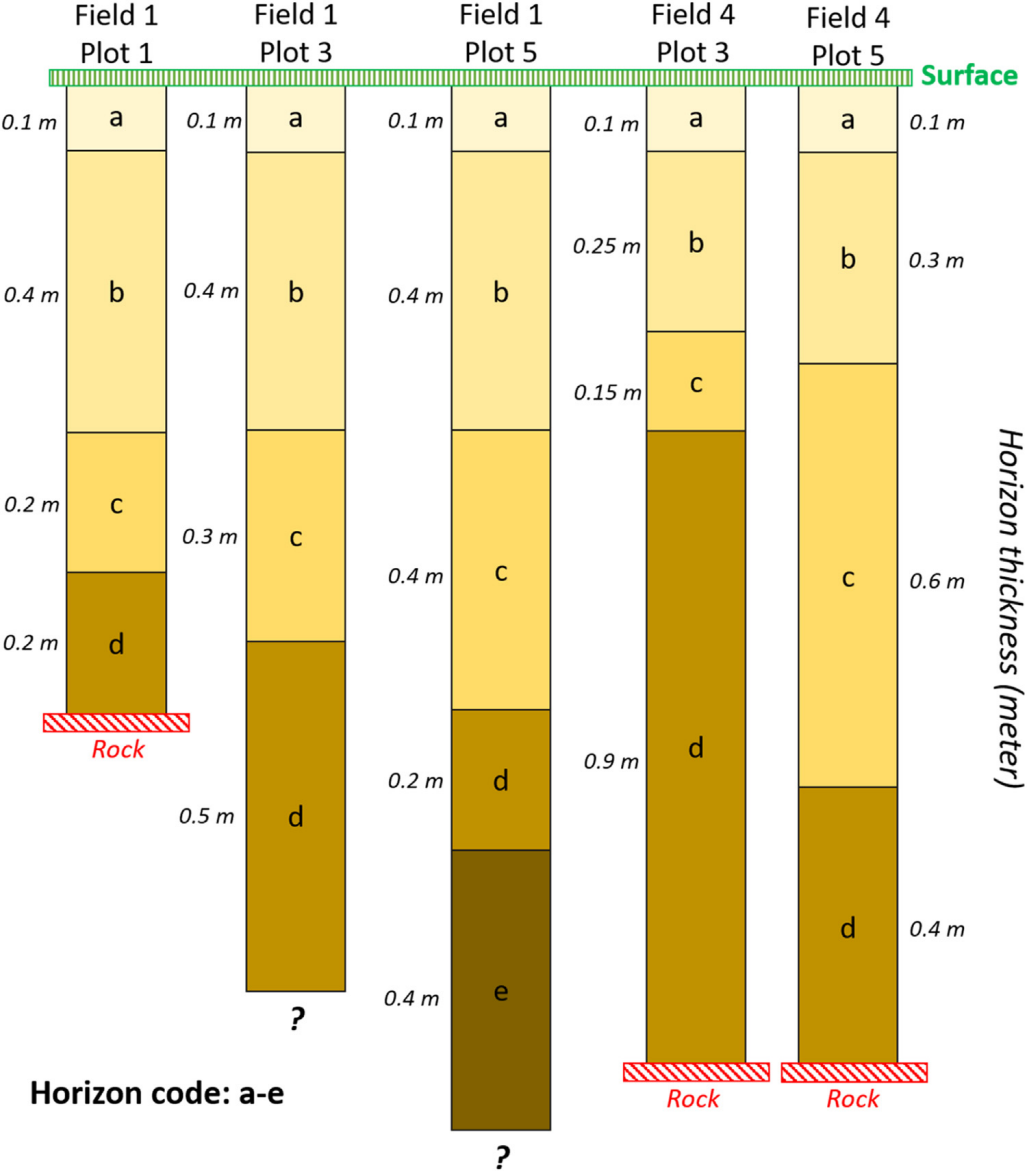
Illustration of soil horizons in the 5 plots where soil pits were created.

A total of 5 soil pits were excavated in Fields N°1 and N°4, each approximately 1 m² in surface area. These pits were located between two vine rows, at the center of a measurement plot. The depth of exploration varied depending on the hardness of the deeper soil horizons (on average, the soil depth was greater than 1 m). In 2 plots (plot 3 & 5 in field N°1), it was assumed that the maximum soil depth was greater than the excavation depth, because the rock horizon was not found. In each plot, soil horizons were identified, which are illustrated in Fig. 7.

Variables presented in Table 4: THICKNESS, FIELD_CAPACITY, PERM_WILT_POINT, BULK_DENSITY and ROCK_FRAGMENT were measured for each horizon. THICKNESS was measured in field with a tape ruler. To determine FIELD_CAPACITY and PERM_WILT_POINT, the method described by [11] was used, involving the collection of undisturbed soil samples and measuring water retention at specific pressure potentials: −33 kPa for FC and −1500 kPa for PWP. In parallel, BULK_DENSITY was measured using a photogrammetry method, as outlined by [12], which has proven more efficient than traditional core sampling, especially in stony soils. The software Agisoft Metashape® was employed for photogrammetry. The ROCK_FRAGMENT was quantified following the procedure of [13], which involved sieving soil samples to isolate particles larger than 2 mm in diameter, and calculating their gravimetric ratio after drying at 105 °C for 48 h. These soil parameters were used to calculate soil available water capacity (SAWC) for each soil horizon (denoted by i), using the equation provided by [14] by Equation 1.

## Limitations

The main limitation of this dataset is that it was collected from a single vineyard over one year. The vineyard had specific characteristics, such as plant density and training system. As a result, this dataset is notably suitable for applications in other Mediterranean rain-fed vineyards with similar field settings. However, caution is strongly advised when generalizing the relationships between vine water status indicators to other vineyards with different field conditions. Additionally, the 2022 vintage in the study region was particularly dry, which may affect the relationships between the data. Yet, this specific condition offers an ideal condition for revealing a wide range of vine water status variation, which is suitable for modeling. In other climatic years with less water deficit, the relationships between variables could be susceptible for changes. Lastly, viticultural practices, such as the use of inter-row crops or canopy trimming, can influence vine shoot growth dynamics. Yet, since most of the fields in this dataset were managed similarly, it limits the ability to assess the impact of different farming practices on the relationships between the collected data.

## Ethics Statement

The current work does not involve human subjects, animal experiments, or any data collected from social media platforms. Therefore, the authors declare that there are no ethical issues with the data presented.

## CRediT Author Statement

**Yulin Zhang:** Conceptualization, Methodology, Investigation, Writing – original draft; **Léo Pichon:** Conceptualization, Writing – review & editing; **Bruno Tisseyre:** Writing – review & editing, Supervision.

## Data Availability

ZenodoA dataset of three vine water status indicators, weather records and soil available water capacity components collected from a rain-fed Mediterranean vineyard (Original data).
